# Communal nesting differentially attenuates the impact of pre-weaning social isolation on behavior in male and female rats during adolescence and adulthood

**DOI:** 10.3389/fnbeh.2023.1257417

**Published:** 2023-10-17

**Authors:** Jessica Bratzu, Maria Ciscato, Augusta Pisanu, Giuseppe Talani, Roberto Frau, Patrizia Porcu, Marco Diana, Fabio Fumagalli, Patrizia Romualdi, Laura Rullo, Viviana Trezza, Roberto Ciccocioppo, Fabrizio Sanna, Liana Fattore

**Affiliations:** ^1^Neuroscience Institute, National Research Council of Italy (CNR), Cagliari, Italy; ^2^Department of Biomedical Sciences, University of Cagliari, Monserrato, Italy; ^3^G.Minardi’ Cognitive Neuroscience Laboratory, CPMB Science Department, University of Sassari, Sassari, Italy; ^4^Department of Pharmacological and Biomolecular Sciences ‘Rodolfo Paoletti’, University of Milan, Milan, Italy; ^5^Department of Pharmacy and Biotechnology, University of Bologna, Bologna, Italy; ^6^Department of Science, University “Roma Tre”, Rome, Italy; ^7^School of Pharmacy, Center for Neuroscience, Pharmacology Unit, University of Camerino, Camerino, Italy

**Keywords:** early-life stress, social enrichment, isolation, communal nesting, anxiety-like behaviors, marble-burying, pre-pulse inhibition, sex-difference

## Abstract

**Introduction:**

Early social isolation (ESI) disrupts neurodevelopmental processes, potentially leading to long-lasting emotional and cognitive changes in adulthood. Communal nesting (CN), i.e., the sharing of parental responsibilities between multiple individuals in a nest, creates a socially enriching environment known to impact social and anxiety-related behaviors.

**Methods:**

This study examines the effects of (i) the CN condition and of (ii) ESI during the 3^rd^ week of life (i.e., pre-weaning ESI) on motor, cognitive, and emotional domains during adolescence and adulthood in male and female rats reared in the two different housing conditions, as well as (iii) the potential of CN to mitigate the impact of ESI on offspring.

**Results:**

We found that in a spontaneous locomotor activity test, females exhibited higher activity levels compared to males. In female groups, adolescents reared in standard housing (SH) condition spent less time in the center of the arena, suggestive of increased anxiety levels, while the CN condition increased the time spent in the center during adolescence, but not adulthood, independently from ESI. The prepulse inhibition (PPI) test showed a reduced PPI in ESI adolescent animals of both sexes and in adult males (but not in adult females), with CN restoring PPI in males, but not in adolescent females. Further, in the marble burying test SH-ESI adolescent males exhibited higher marble burying behavior than all other groups, suggestive of obsessive-compulsive traits. CN completely reversed this stress-induced effect. Interestingly, ESI and CN did not have a significant impact on burying behavior in adult animals of both sexes.

**Discussion:**

Overall, our findings (i) assess the effects of ESI on locomotion, sensorimotor gating, and compulsive-like behaviors, (ii) reveal distinct vulnerabilities of males and females within these domains, and (iii) show how early-life social enrichment may successfully counteract some of the behavioral alterations induced by early-life social stress in a sex-dependent manner. This study strengthens the notion that social experiences during early-life can shape emotional and cognitive outcomes in adulthood, and points to the importance of social enrichment interventions for mitigating the negative effects of early social stress on neurodevelopment.

## 1. Introduction

Traumatic events, occurring early in life, are known to enhance the vulnerability to develop brain diseases in adulthood (Sarkar et al., 2019; Hegde and Mitra, 2020; Palma-Gudiel et al., 2020). To evaluate the vast array of long-lasting alterations after the exposure to various stress paradigms in the early post-natal life, numerous animal models have been developed and validated over the years (Murthy and Gould, 2018). Several changes induced by early-life stress have been described, including impairments in memory formation and cognitive performances (Reincke and Hanganu-Opatz, 2017; Talani et al., 2023), enhanced anxiety level (Brunton, 2015), depression-like symptoms (Vetulani, 2013) and vulnerability to develop drug use disorders (Delavari et al., 2016; de Almeida Magalhães et al., 2017).

In rodents, the neonatal maternal separation is one of the most commonly used models of early-life stress exposure to study the consequent alterations at the endocrine, neurophysiological, and behavioral level (Nishi et al., 2014; Mejía-Chávez et al., 2021). In this model, pups are removed from the dam’s nest for a few hours each day during the first two weeks of postnatal life, although protocols may differ in terms of length of separation or time window in which they are applied (Plotsky and Meaney, 1993; Hall, 1998; Fabricius et al., 2008; Cirulli et al., 2009; Marco et al., 2009; Bailoo et al., 2014). Interestingly, impairments are typically observed in male but not female rodents, which highlights a sex-dependent effect induced by this environmental manipulation (Romeo et al., 2003; Mehta and Schmauss, 2011; Kundakovic et al., 2013; Bailoo et al., 2014; Talani et al., 2023). However, other studies report opposite results (Veenema et al., 2007; Tsuda and Ogawa, 2012; Cui et al., 2020), thereby suggesting that the influence of sex as a factor for vulnerability to the effects of maternal separation needs further investigation and clarification. Age is another factor that may significantly influence the effect of early-life social experiences, as both social isolation and enrichment during the first three postnatal weeks may affect behavior differently in adolescent and adult rats (Macrì et al., 2010; Daoura et al., 2011).

Pre-weaning social isolation provides a further experimental animal model of early-life stress. This model is much less characterized and implies a shorter time window and duration of pups’ separation. Such a pre-weaning social stress significantly enhances locomotor activity in a novel environment in male and female adolescent rats and increases the response to low-to-moderate doses of methamphetamine (Pritchard et al., 2012), suggesting that subjects with a history of early-life social stress may be particularly vulnerable to the effects of psychostimulants. Notably, this latter effect occurred at different doses of methamphetamine for male (3.0 mg/kg) and female (1.0 mg/kg) rats, revealing a sex-dependent effect of early social isolation (ESI) on vulnerability to psychostimulants effects (Pritchard et al., 2012). Other studies demonstrated that early-life social isolation resulted in impulsive behavior (as indexed by faster movements) in adolescent rats and that the effect was more pronounced in males than females, suggesting that adolescent female rats are more resistant to the effects of early social stress on motor impulsive behavior (Pritchard et al., 2012). Yet, early social isolation may also lead to hypoactive behaviors in rats along with greater propensity to show depressive-like responses to stress (Spivey et al., 2009).

In rodents, the behavioral endophenotype most altered by peri-weaning social isolation is the prepulse inhibition (PPI) of the startle reflex (Wilkinson et al., 1994; Varty and Higgins, 1995; Frau et al., 2015; Sun et al., 2021). PPI refers to the inhibition of the startle response by presentation of a weak intensity prestimulus or prepulse (acoustic or tactile) that immediately precedes the pulse (startle) stimulus (Swerdlow et al., 1995). This paradigm provides a well-established operational measure of sensorimotor gating, the process that the brain enacts to attribute the salient aspects of sensory information from the environmental stimuli. As such, this protective mechanism gates out irrelevant and/or redundant sensory stimulation, thereby preventing information overload and preserving cognitive integration with its related neurobehavioral outcomes.

Another behavior affected by neonatal maternal separation is burying behavior, defined as a physiologic process, in laboratory rodents, that consists in burying harmless objects (e.g., glass marbles) using bedding material. When burying behavior become excessive, however, it is considered a valid prediction of perseveration/compulsion related disorders (Taylor et al., 2017). Recent studies have shown that mice exposed to maternal separation showed a significant increase in the number of buried marbles as clear increase of perseverative behavior, providing further empirical support for a link between childhood adversity and development of impulsive/compulsive traits (Ou et al., 2021; Jarrar et al., 2022). Interestingly, sex-dependent differences have been reported in the marble burying test, although with discrepant results (Burke et al., 2016; Mancini et al., 2021; Emtyazi et al., 2022).

If early-life social deprivation has been associated to enhanced risk of altered behavior and vulnerability to mental diseases, a potential positive impact of early social enrichment on behavior and brain development has also been described (Cirulli et al., 2010). An ethological paradigm to create a pre-weaning complex social environment is represented by communal nesting (CN), i.e., the sharing of caregiving behaviors among a group of dams in a single nest (Branchi, 2009). CN mirrors the developing pups’ natural rearing setting, which is typically much richer than the standard laboratory conditions (Branchi et al., 2006). CN has been reported to affect anxiety and depressive-like behaviors in mice (Branchi and Alleva, 2006), stimulate social interaction (D’Andrea et al., 2007), and increase resiliency to social stress in male mice (Branchi et al., 2013). Whether CN condition can attenuate, if not fully prevent, the behavioral alterations induced by an early-life stress in adolescent and/or adult rats remains to be investigated.

Here, we explored, for the first time, the effects of CN on an early social isolation (ESI) protocol, milder than the classical maternal separation paradigm, as it consisted in depriving pups of any social contact from postnatal day (PND) 14 to PND21 for 30 min/day only. Specifically, this study was undertaken to assess whether during adolescence and/or adulthood (i) ESI alters motor activity, obsessive-compulsive behavior and sensorimotor gating in rats, (ii) CN *per sé* is able to affect animals’ behavioral performances, (iii) CN can reverse the potential negative impact of ESI on motor activity, obsessive-compulsive behavior and sensorimotor gating. Moreover, in light of the sexual dimorphism in the “emotional brain” circuits (Fone and Porkess, 2008; Pallayova et al., 2019) and the sex-dependent differences in the neural response to social behaviors (Zilkha et al., 2021) and stress (Oyola and Handa, 2017), this study also evaluated (iv) whether early-life social rearing conditions affect behaviors of adolescents and adults in a sex-dependent manner. The Wistar rat strain was selected in light of its use in previous studies investigating either the effects of early-life social isolation on behavior and potential nonpharmacological interventions to mitigate its behavioral effects (Daoura et al., 2010; Lundberg et al., 2016; Khalifeh et al., 2019; Mohammadian et al., 2019), including the CN paradigm (Uriarte et al., 2014).

## 2. Materials and methods

### 2.1. Animals

A total of 64 male (M) and 64 female (F) Wistar rats, born from parents provided by Charles River (Italy) were used for this study, both during adolescence (32 M, 32 F) and early adulthood (32 M, 32 F). Animals were bred at the animal facility of the University of Cagliari (Italy) and housed under a 12/12 h inverted light/dark cycle (light on: 07:00 PM) with constant room temperature (22 ± 2°C) and humidity (60%) and free access to standard laboratory chow (ALTROMIN Diet 1,324, Caipet, Italy) and tap water. Experiments were conducted during the dark phase of the day/night cycle (10:00 AM-1:00 PM). All experimental procedures and animal care were performed in accordance with current Italian legislation (D.L. 26/2014), which requires the Ministry of Health (Rome, Italy) to approve a submitted Research Project for the experimentation on laboratory animals to take place (authorization n. 512/2021-PR). In addition, all procedures were in strict accordance with the European Council Directive on animal use in research (n. 2010/63/EU). All efforts were made to minimize animal discomfort and suffering to reduce the number of animals used, in compliance with the ARRIVE guidelines (Percie du Sert et al., 2020). In all the behavioral test, each trial included animals of the same sex and from different experimental groups, and the order in which animals were tested was balanced across the SH-CTRL, SH-ESI, CN-CTRL and CN-ESI groups, and across the two sexes.

### 2.2. Nesting conditions

Animals were process subjected to one of the following four environmental conditions:

Standard Housing (SH): each female rat was housed with one male in a 42 × 27 × 21 cm Plexiglas cage until pregnancy could be verified. On gestational day 1 (GD1), defined by the detection of spermatozoa in the vaginal smears, the male rat was removed from the cage and the pregnant dam was housed in an individual cage and left undisturbed till delivery. Litters were culled to 8 animals (4 M, 4 F) within 24 h from birth, and left undisturbed till weaning.
Communal Nesting (CN): 3 females were housed with 1 male, which was removed after 16 days. The group of dams remained undisturbed till delivery. Each litter was culled to 8 animals (4 M, 4 F) within 24 h from birth, and left undisturbed till weaning.
Standard Housing + Early Social Isolation (SH + ESI): female rats were mated and housed as described in SH. After birth (PND1), the litters were left undisturbed with their mothers until PND14, at which time half of the pups from each nest was subjected to an early social isolation (ESI) protocol for 7 consecutive days (i.e., from PND14 to PND21), which consisted of individually removing each pup from the nest for 30 min/day. During the separation period, the pup was placed in a cage with clean bedding.
Communal Nesting + Early Social Isolation (CN + ESI): female rats were mated and housed as described in CN. Half of the pups from each nest was subjected to the ESI from PND14 to PND21, which consisted of individually removing each pup from the nest for 30 min/day. During the separation period, the pup was placed in a cage with clean bedding.

**Figure 1:**
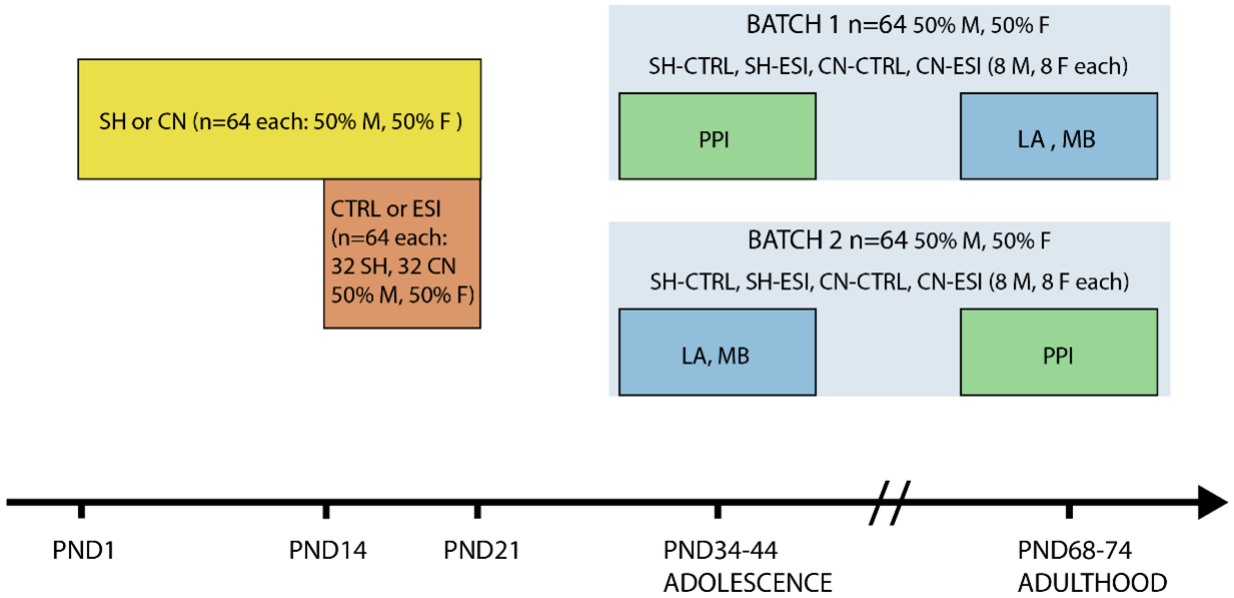
Timeline of social manipulation and behavioral testing. Rats (*n* = 128) were subjected to either one of two housing conditions, Standard Housing (SH) or Communal Nesting (CN), until Postnatal day (PND) 21 (*n* = 64 per condition, 50% males and 50% females). Between PND14 and PND21 the animals were exposed to no stress (CTRL) or to an Early Social Isolation (ESI) protocol (*n* = 64 per stress condition, *n* = 32 part of the SH group and *n* = 32 part of the CN group, 50% males and 50% females). Afterward, half of the rats in each environmental condition (Housing x Stress) was tested in a Pre-Pulse Inhibition test (PPI) in adolescence and in the Locomotor Activity test (LA) and Marble Burying test (MB) in adulthood (Batch 1), the remaining half was tested in LA and MB in adolescence and in PPI in adulthood (Batch 2).

Animals were disturbed as little as possible during mating and breeding. After weaning (PND21), animals were housed in same sex/environmental condition groups of 3 or 4 per cage and tested both during adolescence and early adulthood. A first batch of animals underwent the PPI test during adolescence (i.e., between PND38 and PND40), and the locomotor activity test and the marble burying test during adulthood (i.e., between PND68 and PND78). The second batch of animals was tested in the locomotor activity test and the marble burying test during adolescence (i.e., between PND34 and PND44), and in the PPI test during adulthood (i.e., between PND72 and PND74). Each of the eight experimental groups, four per batch, included both male (*n* = 8) and female (*n* = 8) rats (Figure 1). Before starting the experiments, rats were handled daily for 5 min for 3 days by the same researchers who performed the experiments.

### 2.3. Spontaneous locomotor activity

Rats were individually tested for motor activity under standardized environmental conditions (in a soundproof room under a 30 lx dim light) with a Digiscan Animal Activity Analyzer (Omnitech Electronics, Columbus, Ohio), as previously described (Struik et al., 2017). Each cage (42 × 42 × 63 cm) had two sets of 16 photocells located at right angles to each other, projecting horizontal infrared beams 2.5 cm apart and 2 cm above the cage floor and a further set of 16 horizontal beams whose height was adapted to the size of the animals. Horizontal and vertical activity were measured as total number of sequential infrared beam breaks (counts) in the sensors, while center activity was recorded as the time spent by rats in the central part of the arena, beginning immediately after placing the animals into the cage, over a period of 60 min.

### 2.4. Pre-pulse inhibition (PPI) of the acoustic startle reflex

PPI of the startle reflex was measured as previously described (Spano et al., 2010; Marti et al., 2021). Briefly, the apparatus (Med Associates, St Albans, VT, United States) consisted of 4 standard cages placed in sound-attenuated chambers with fan ventilation. Each cage consisted of a Plexiglas cylinder of 9 cm diameter, mounted on a piezoelectric accelerometric platform connected to an analog-digital converter. Two separate speakers conveyed background noise and acoustic bursts, each one properly placed so as to produce a variation of sound within 1 dB across the startle cage. Both speakers and startle cages were connected to a main PC, which detected and analyzed all chamber variables with specific software. Before each testing session, acoustic stimuli and mechanical responses were calibrated *via* specific devices supplied by Med Associates. A background noise of 70 dB was delivered for the entire PPI session, including the acclimation period of 5 min. Acclimation was followed by three consecutive sequences of trials (1^st^, 2^nd^ and 3^rd^ block). Unlike the 1^st^ and the 3^rd^ block, during which rats were presented with only five pulse-alone trials of 115 dB, the 2^nd^ block consisted of a pseudorandom sequence of 50 trials, including 12 pulse-alone trials, 30 trials of pulse preceded by 74, 78, or 86 dB pre-pulses (10 for each level of pre-pulse loudness), and 8 no-stimulus trials, where only the background noise was delivered. Inter-trial intervals were selected randomly between 10s and 20s. PPI values were calculated as percentage using the following formula:


$$\mathrm{100}-\left[\left(\begin{array}{l} \mathrm{mean startle amplitude for}\;\mathrm{pre}-\mathrm{pulse pulse trials}/\\ \mathrm{mean startle amplitude for pulse alone trials} \end{array}\right)*\mathrm{100}\right]$$

### 2.5. Marble burying test

The marble burying test was performed in open transparent Plexiglas boxes (54 × 34.5 × 20 cm) and under a dim light (30 lux), as previously described (Zanda et al., 2017; Costa et al., 2019). The box floor was covered with 5 cm of bedding, on which 24 glass marbles (diameter: 1.2 cm) were positioned arranged in 6 rows of 4 marble each. Animals were allowed to acclimatize to the test room for 15 min before starting the test. At the beginning of the test session, each animal was placed in a marble-free area of the test cage (34.5 × 15 cm) and allowed to freely explore the cages for 30 min, while a video camera located on the room ceiling monitored the animal’s activity. At the end of the session, each subject was gently removed from the box and the number of marbles totally (>95%) buried was counted by an experimenter blind to the experimental conditions. Bedding was changed after each session, and marbles cleaned with soap and tap water to avoid any olfactory cue.

### 2.6. Statistical analyses

A sample size calculation *via* the G*Power software (G*Power 3.1.9.2), was performed to assess the required minimum number of rats to be employed in the study. A critical effect size f2 = 0.35 was chosen based on pilot experiments, with 80% power (1-β = 0.80) and a 5% significance level (α = 0.05) for each investigated behavior in an ANOVA.

Data are expressed as mean ± standard error of the mean (SEM) of 8 rats/group. Shapiro Wilk’s test and Levene’s test were carried out to assess the normal distribution of the dependent variables and homogeneity of variances, respectively. Afterward, a three-way analysis of variance (ANOVA) was run with the three between subject factors: Sex (Male, Female), Housing (Nesting, Standard) and early social stress (CTRL, ESI). Independent three-way ANOVAs were performed for adolescent and adult rats. When the ANOVA revealed significant first or second order interactions or main effects of factors, Bonferroni’s corrected pairwise comparisons were conducted to inspect significant differences between experimental groups. The capital letters reported in the Figures refer to significant main effects and/or interactions between factors while symbols on bars refer to the significant differences in direct comparisons between experimental groups (i.e., simple effects).

All the analyses were carried out using SPSS (IBM Corp. in Armonk, NY). A value of *p* < 0.05 was considered statistically significant.

## 3. Results

### 3.1. Adolescents

#### 3.1.1. Locomotor activity

**Figure 2:**
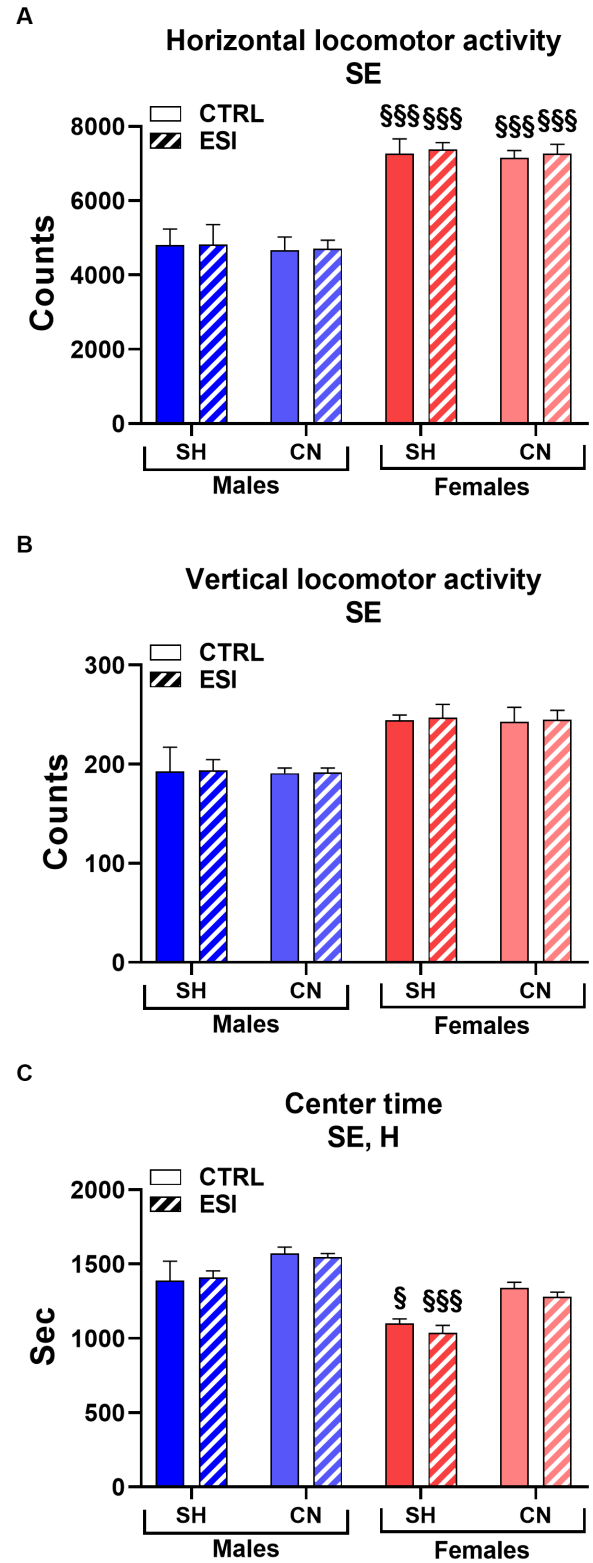
Horizontal **(A)**, vertical **(B)**, and center **(C)** locomotor activity in adolescent male (*blue*) and female (*red*) rats. Data are expressed as means ± SEM of the total number of counts **(A,B)** and the total time spent in the central part of the arena **(C)**. SH, standard housing, CN, communal nesting, CTRL, no stress, ESI, early social stress. Three-way ANOVA followed by Bonferroni’s pairwise comparisons (*n* = 8 rats/group). SE, significant effect of sex; H, significant effect of housing. ^§^*p* < 0.05; ^§§§^*p* < 0.001 vs. the respective male group.

As shown in Figure 2, in adolescent males and females the spontaneous locomotor activity test revealed a significant main effect of sex in all three parameters considered, i.e., horizontal and vertical activity and the time spent in the center of the arena (F_1, 56_ = 106.33, F_1, 56_ = 34.66 and F _1, 56_ = 49.25, all *p* < 0.0001, for horizontal and vertical activity, and center time, respectively). In particular, female rats displayed greater horizontal (+53%, averaged mean of all female groups vs. averaged mean of all male groups, *p* < 0.0001, Figure 2A), and vertical (+27%, averaged mean of all female groups vs. averaged mean of all male groups, *p* < 0.0001, Figure 2B) activity and spent less time (−12%, averaged mean of all female groups vs. averaged mean of all male groups, p < 0.0001, Figure 2C) in the center of the arena compared to males, regardless of the housing conditions and the presence of ESI.

Interestingly, a significant main effect of Housing (F_1, 56_ = 23.46, *p* < 0.0001) was observed for the time spent in the center of the arena, indicating that rats reared in the CN condition spent more time in the center of the arena compared to rats reared in SH conditions (+9%, averaged mean of all CN groups vs. averaged mean of all SH groups, *p* < 0.0001, Figure 2C) regardless of sex or previous exposure to the ESI protocol.

By contrast, no significant main effects of stress nor first or second order interactions between factors (i.e., Housing, Stress, Sex) were observed in the three parameters considered.

Finally, Bonferroni’s pairwise comparisons carried out on simple effects confirmed that all the female groups displayed greater horizontal activity values when compared to their matched male groups (all *p* < 0.001, Figure 2A) and revealed that both SH female groups spent less time in the center of the arena compared to their matched male groups (*p* < 0.05 and *p* < 0.001 for CTRL and ESI groups, respectively, Figure 2C).

#### 3.1.2. Pre-pulse inhibition (PPI) test

**Figure 3:**
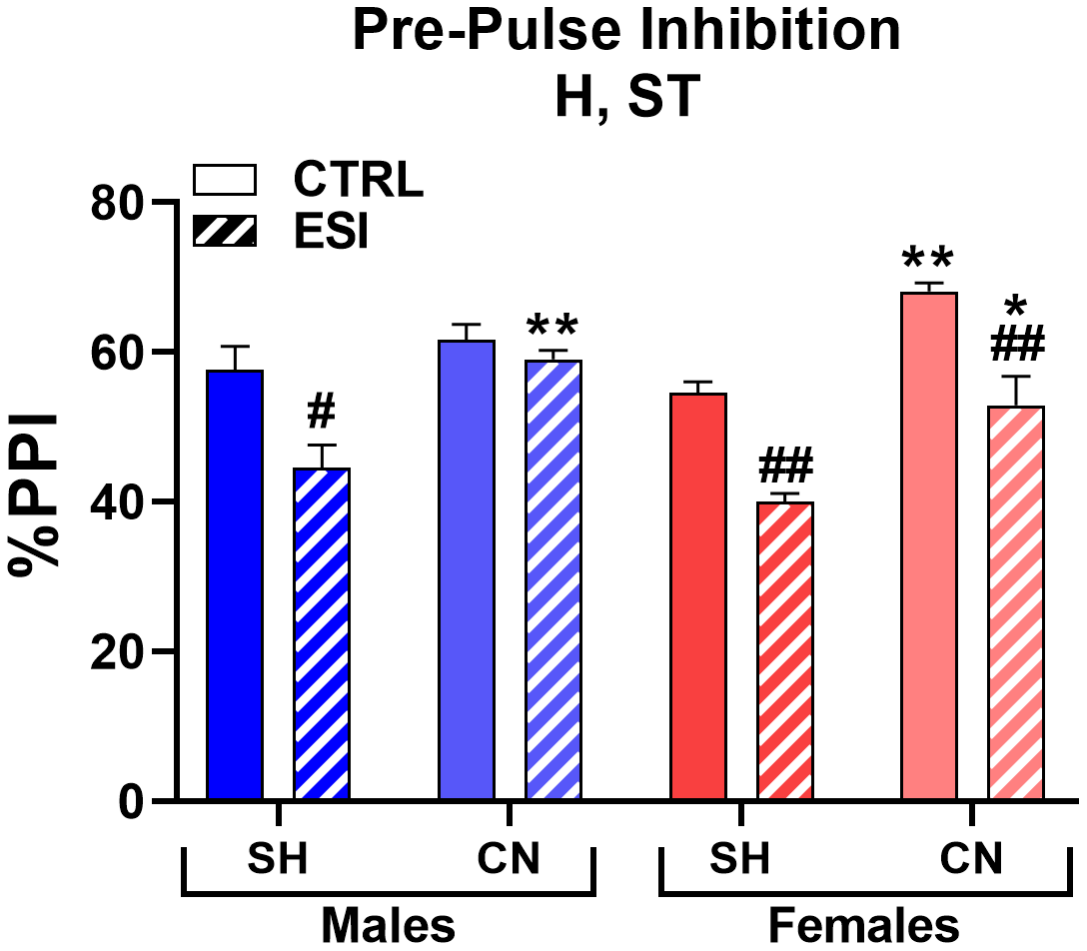
Pre-pulse inhibition (PPI) test in adolescent male (*blue*) and female (*red*) rats. Data are expressed as means ± SEM of the %PPI response over the 3 pre-pulse intensities. SH: standard housing, CN, communal nesting, CTRL, no stress, ESI, early social stress. Three-way ANOVA followed by Bonferroni’s pairwise comparisons (*n* = 8 rats/group). H, significant effect of housing; ST, significant effect of stress; SE*ST, significant sex x stress interaction. ^*^p < 0.05, ^**^p < 0.01 vs. the respective SH group; ^#^*p* < 0.05, ^##^*p* < 0.01 vs. the respective CTRL group.

When adolescent rats were tested for sensorimotor gating, three-way ANOVA (factors: Housing, Stress, Sex) yielded significant main effects for Housing (F_1, 56_ = 41.347, *p* < 0.0001) and Stress (F_1, 56_ = 42.369, *p* < 0.0001), but not for Sex or interactions (Figure 3). Accordingly, Bonferroni’s comparisons on main effects shown that the CN condition was associated with a significantly higher PPI (60.4%, averaged mean of all CN groups vs. 49.2%, averaged mean of all SH groups), with an average increase of 11.2% (*p* < 0.001), with respect to the SH condition, regardless of sex or ESI; likewise, ESI was associated to a lower PPI (49.1%, averaged mean of all ESI groups vs. 60.5%, averaged mean of all CTRL groups), regardless of the Housing condition (−11.4%, *p* < 0.001).

Moreover, pairwise comparisons carried out on simple effects showed a lower PPI in SH-ESI groups when compared to the respective SH-CTRL controls (males: −13.6%, *p* < 0.05 and females: −14.6%, *p* < 0.01). Notably, although no significant three-way interaction was detected, pairwise comparisons suggested that CN may prevent the stress effect in males but not females. Accordingly, PPI in males subjected to CN did not differ between CN-ESI and CN-CTRL (59.0% vs. 61.6%, respectively); by contrast, in females a significant difference between CN-CTRL and CN-ESI was detected (68.1% vs. 52.9%, respectively, *p* < 0.01). Finally, both CN-CTRL and CN-ESI females shown greater PPI compared to their SH matched groups (CTRL: +13.5%, *p* < 0.01; ESI: +12.9%, *p* < 0.05).

#### 3.1.3. Marble burying test

**Figure 4:**
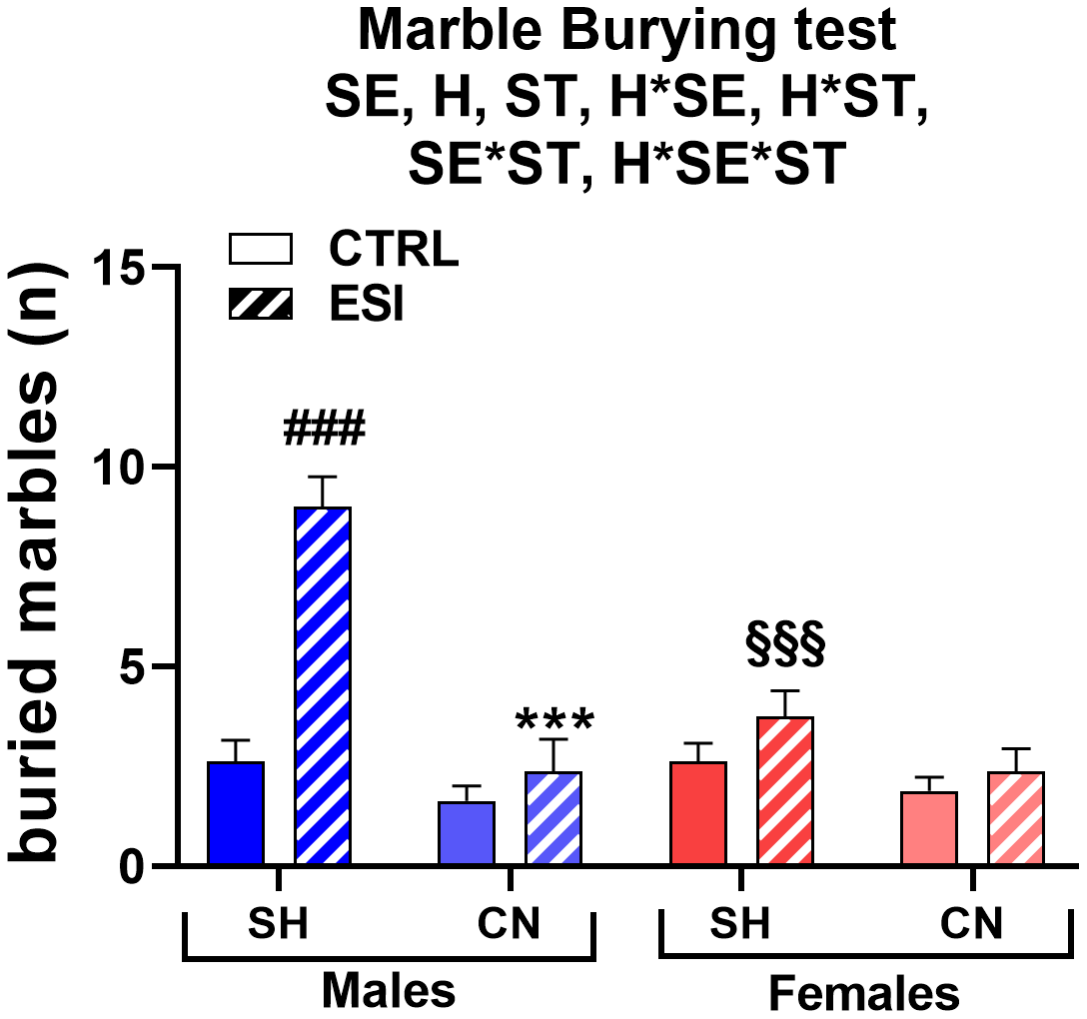
Marble Burying test in adolescent male (*blue*) and female (*red*) rats. Values are means ± SEM of the number of marbles entirely covered with bedding. SH, standard housing, CN, communal nesting, CTRL, no stress, ESI, early social stress. Three-way ANOVA followed by Bonferroni’s pairwise comparisons (*n* = 8 rats/group). SE, significant effect of sex; H, significant effect of housing; ST, significant effect of stress; H*SE, significant housing x sex interaction; H*ST, significant housing x stress interaction; SE*ST significant sex x stress interaction; H*SE*ST, significant housing x sex x stress interaction. ^***^p < 0.001 vs. the respective SH group; ^###^*p* < 0.001 vs. the respective CTRL group; ^§§§^*p* < 0.001 vs. the respective male group.

In the marble burying test, a sex-dependent effect of ESI was observed in adolescent rats housed under SH conditions, being the number of buried marbles increased in SH-ESI males but not females (Figure 4). Notably, the CN condition prevented the effect induced by the ESI protocol. Three-way ANOVA yielded all significant main effects and interactions, including a three-level interaction (Sex: F _1, 56_ = 9.22, *p* < 0.004; Housing: F _1, 56_ = 35.08, *p* < 0.0001; Stress: F _1, 56_ = 28.25, *p* < 0.0001; Housing*Sex: F _1, 56_ = 11.16, *p* < 0.001; Housing*Stress: F _1, 56_ = 14.41, *p* < 0.0001; Stress*Sex: F _1, 56_ = 11.16, *p* < 0.001; Housing*Stress*Sex: F _1, 56_ = 9.226, *p* < 0.004).

*Post-hoc* analyses showed that ESI increases the number of buried marbles in adolescent males housed in the SH condition (+242%, *p* < 0.001), whereas females exhibited the same level of burying activity across the CTRL-ESI conditions. Consequently, the performance of SH-ESI groups differed significantly across sexes, with males burying a number of marbles roughly double than that of females (9 ± 0.8 vs. 3.8 ± 0.6, respectively, *p* < 0.001). The CN condition completely reverted the ESI effect in males, with CN-ESI rats burying a lower number of marbles, compared to the SH-ESI ones (−73.6%, *p* < 0.001); further, burying behavior did not significantly differ between CN-ESI and CN-CTRL groups. Conversely, in females, housing condition did not affect burying behavior; accordingly, the burying activity of CN-CTRL and CN-ESI female rats did not differ from that of the SH-CTRL and SH-ESI ones (Figure 4).

### 3.2. Adults

#### 3.2.1. Locomotor activity

**Figure 5:**
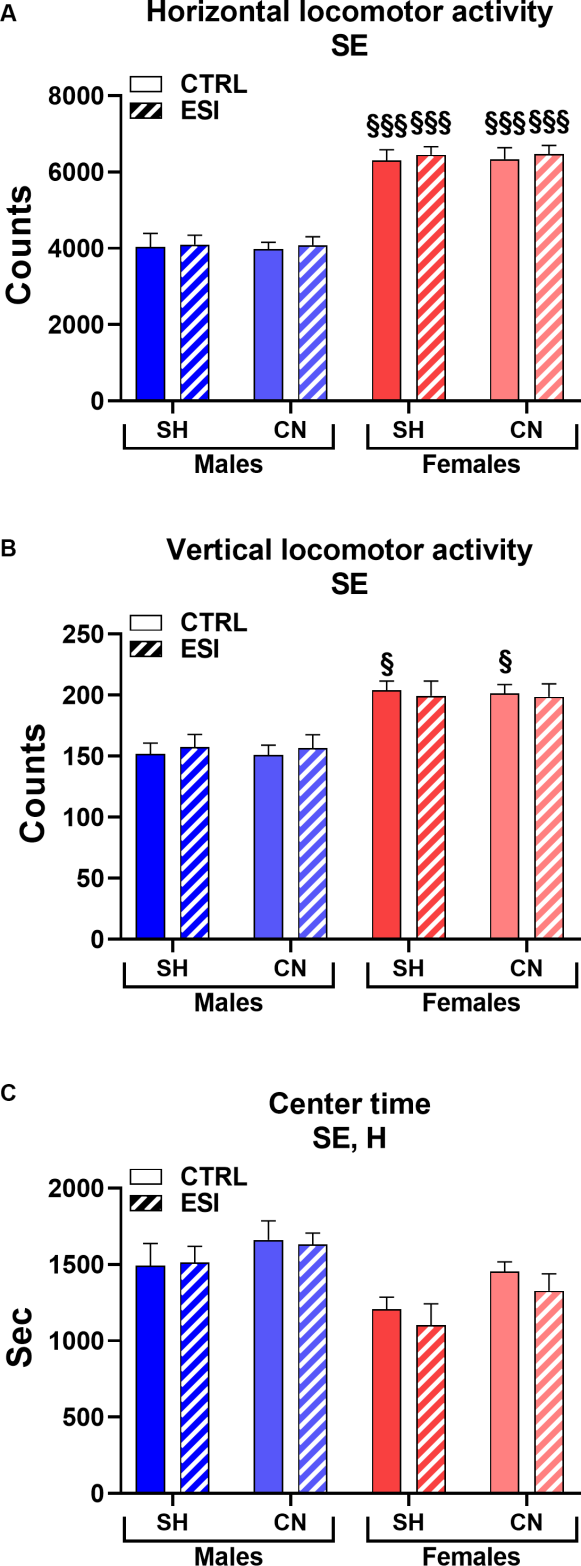
Horizontal **(A)**, vertical **(B)**, and center **(C)** locomotor activity in adult male (*blue*) and female (*red*) rats. Data are expressed as means ± SEM of the total number of counts (*A*, *B*) and the total time spent in the central part of the arena **(C)**. SH: standard housing, CN: communal nesting, CTRL, no stress, ESI, early social stress. Three-way ANOVA followed by Bonferroni’s pairwise comparisons (*n* = 8 rats/group). SE, significant effect of sex; H, significant effect of housing. ^§^*p* < 0.05; ^§§§^*p* < 0.001 vs. the respective male group.

As shown in Figure 5, in adult male and female rats, similarly to adolescents, we detected a significant main effect of Sex in all the three parameters considered in the locomotor activity test (F_1, 56_ = 162.74, F_1, 56_ = 46.13 and F_1, 56_ = 15.22, all *p* < 0.0001, for horizontal and vertical activity, and center time, respectively), indicating that females maintained greater horizontal (+56%, averaged mean of all female groups vs. averaged mean of all male groups, p < 0.0001, Figure 5A) and vertical (+23%, averaged mean of all female groups vs. averaged mean of all male groups, *p* < 0.0001, Figure 5B) activity and spent less time (−15%, averaged mean of all female groups vs. averaged mean of all male groups, p < 0.0001, Figure 5C) in the center of the arena compared to males.

In addition, as seen in adolescent rats, a significant main effect was also observed for the factor Housing (F_1, 56_ = 6.02, *p* < 0.017) indicating that adult rats reared in the CN versus SH condition spent more time in the center of the arena (+9%, Figure 5C) regardless of sex or exposure to the ESI paradigm. By contrast, no significant main effects of Stress nor first or second order interactions between factors were observed in the three parameters considered. Finally, Bonferroni’s pairwise comparisons carried out on simple effects confirmed that all the female groups displayed greater horizontal activity compared to their matched male groups (all *p* < 0.001); similarly, both CTRL female groups (i.e., SH and CN) showed greater vertical activity values compared to their matched male groups.

#### 3.2.2. Pre-pulse inhibition (PPI) test

**Figure 6:**
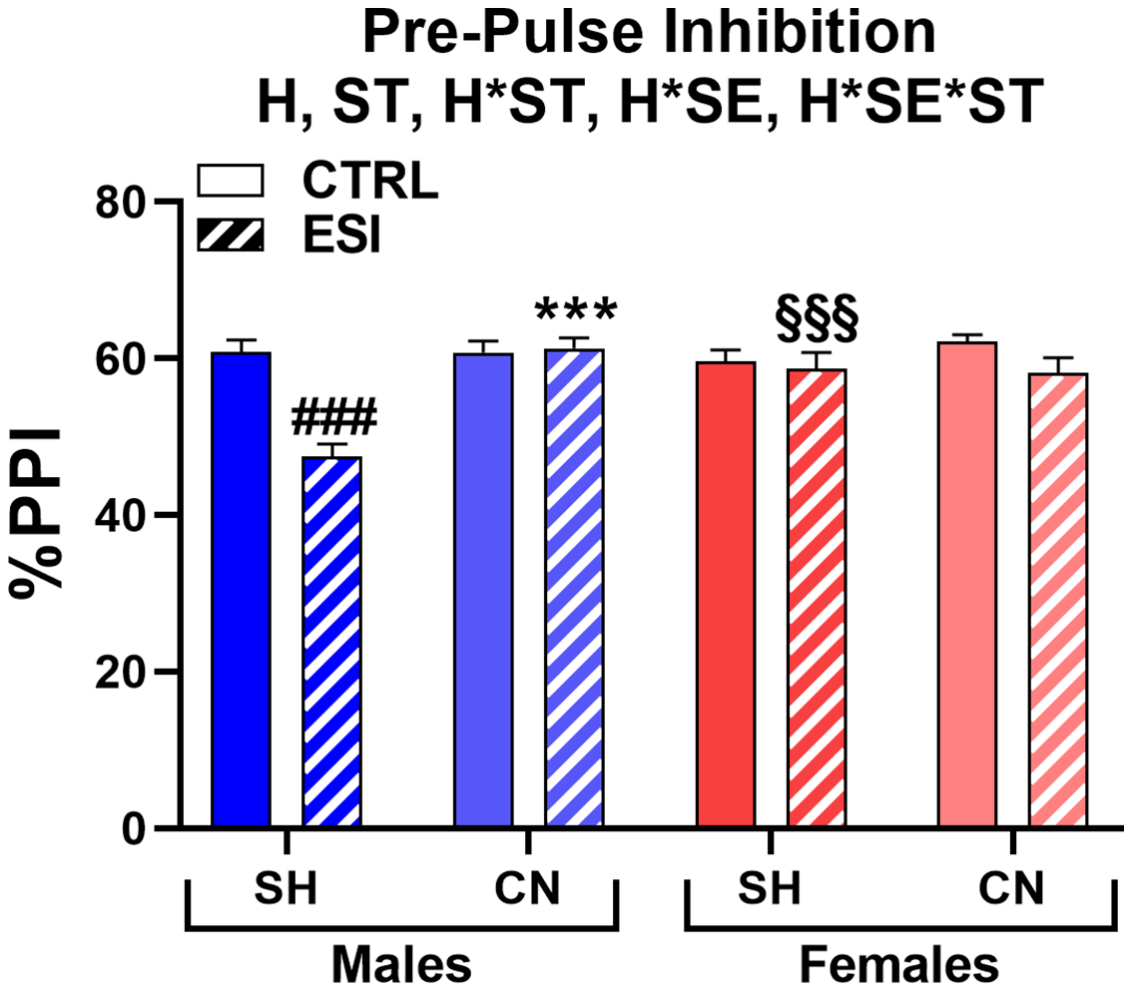
Pre-pulse inhibition (PPI) test in adult male (*blue*) and female (*red*) rats. Data are expressed as means ± SEM of the %PPI response over the 3 pre-pulse intensities. SH: standard housing, CN, communal nesting, CTRL, no stress, ESI, early social stress. Three-way ANOVA followed by Bonferroni’s pairwise comparisons (*n* = 8 rats/group). H, significant effect of housing; ST, significant effect of stress; H*ST, significant housing x stress interaction; H*SE, significant housing x sex interaction; H*SE*ST, significant housing x sex x stress interaction. ^***^p < 0.001 vs. the respective SH group; ^###^*p* < 0.001 vs. the respective CTRL group; ^§§§^*p* < 0.001 vs. the respective sex group.

When adult rats were tested for sensorimotor gating, we found that ESI lowered PPI performance in SH males, an effect fully prevented by the CN condition. Conversely, the ESI protocol had no effect on females (Figure 6). Accordingly, three-way ANOVA yielded main effects for Housing (F_1, 56_ = 12.357, *p* < 0.001) and Stress (F_1, 56_ = 15.892, *p* < 0.0001), significant first order interactions for Housing*Sex (F_1, 56_ = 6.810, *p* < 0.05) and Housing*Stress (F_1, 56_ = 5.956, *p* < 0.05), as well as a significant second order interaction Housing*Stress*Sex (F_1, 56_ = 14.294, *p* < 0.0001).

*Post-hoc* analyses showed that SH-ESI male rats had a significantly lower PPI (−13.3%) when compared to the respective SH-CTRL group (47.5% vs. 60.8%, *p* < 0.001). Interestingly, CN abolished the ESI-induced decrease in PPI (61.3% vs. 47.5%, *p* < 0.001, Figure 6) to the point that a difference between ESI and CTRL male rats was no longer evident. By contrast, neither stress nor housing condition influenced the PPI score in females. As a result, a sex-related difference was also detected, with SH-ESI males showing a significantly lower PPI (−11.2%) compared to the corresponding female group (47.5% vs. 58.7%, p < 0.001, Figure 6).

#### 3.2.3. Marble burying test

**Figure 7:**
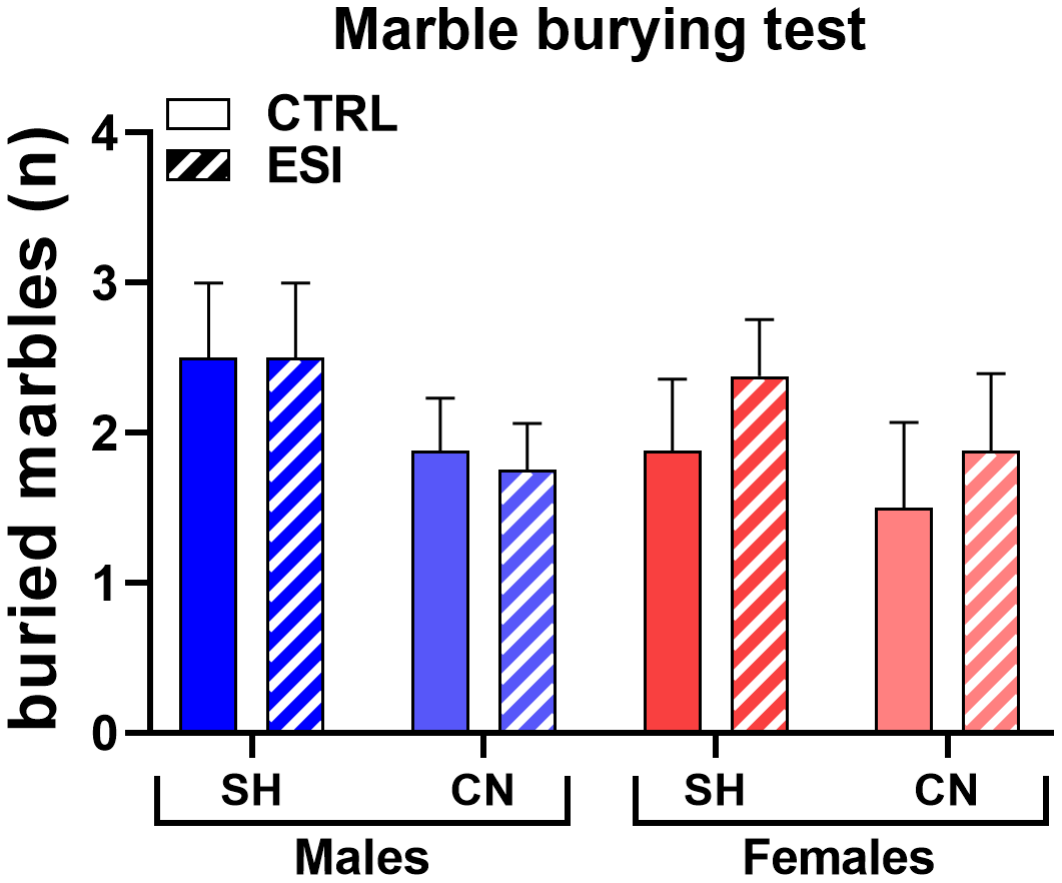
Marble Burying test in adult male (*blue*) and female (*red*) rats. Values are means ± SEM of the number of marbles entirely covered with bedding. SH, standard housing, CN, communal nesting, CTRL, no stress, ESI, early social stress. Three-way ANOVA yielded not significant effects (*n* = 8 rats/group).

At variance from what observed in adolescent rats, burying behavior in adults displayed no significant differences across experimental groups, as neither housing condition nor early stress or sex exerted a significant effect on the number of fully buried marbles (Figure 7). Three-way ANOVA (factors: Housing, Stress, Sex) yielded no significant main effects or interactions (F_1, 56_ = 0.000, *p* = 1.00).

## 4. Discussion

**Figure 8:**
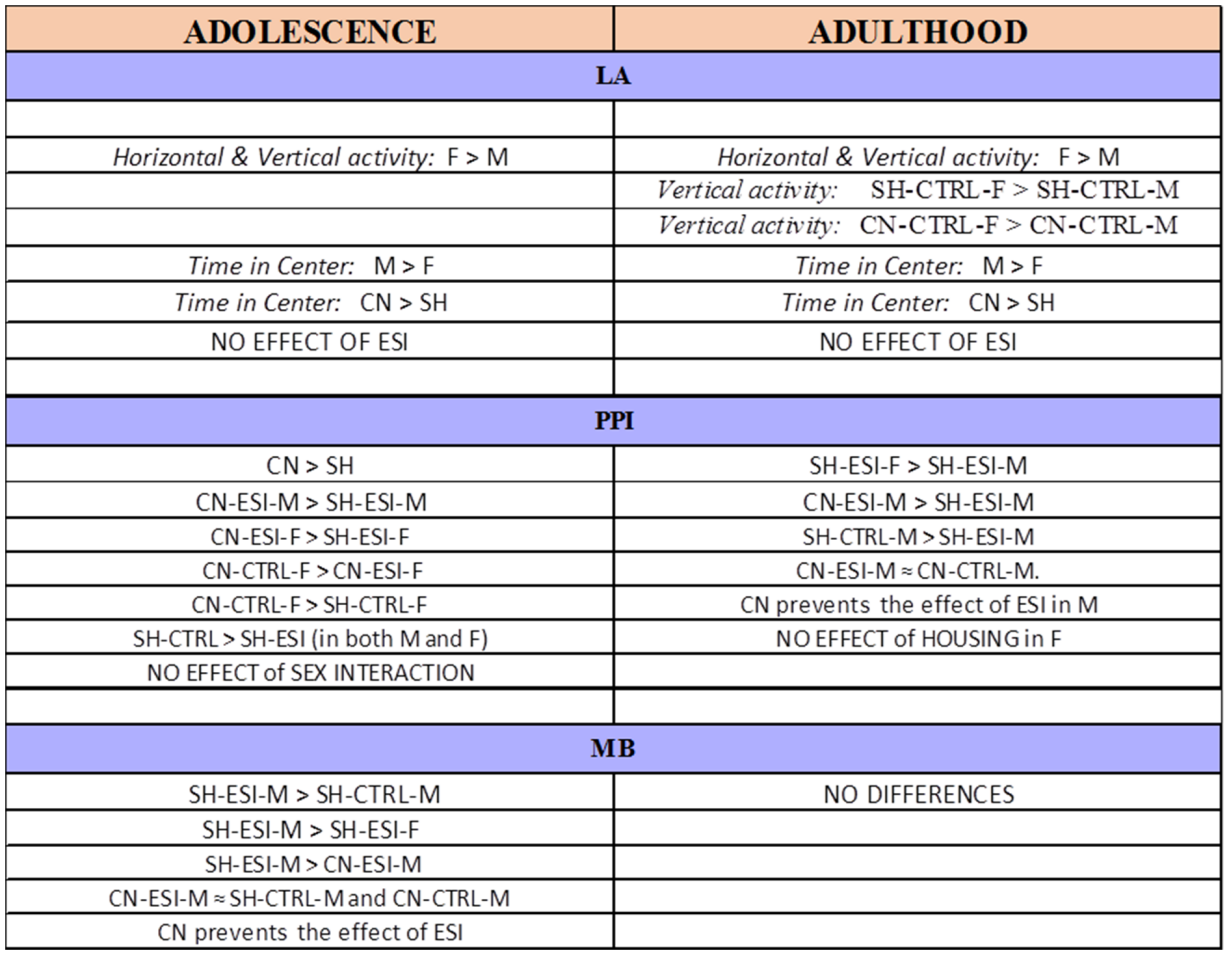
Summary of the results obtained in adolescent and adult male (M) and female (F) rats. CN, Communal Nesting, CTRL, No Stress, ESI, Early Social Isolation, LA, Locomotor Activity test, MB, Marble Burying test, PPI, Pre-Pulse Inhibition test, SH, Standard Housing.

Early-life experiences, including parental care and social interactions, have profound impact on brain development and subsequent behavioral expression. In rodents, pre-weaning social isolation has been associated with behavioral alterations in the offspring that manifest during adolescence and may persist into adulthood (Hall, 1998). This study tested the hypothesis that an early-life socially enriched environment, such as that provided by the communal nesting condition, may attenuate (if not fully prevent) the behavioral effects induced by pre-weaning social isolation in male and female rats. Results revealed that early-life social stress (i.e., pre-weaning isolation) induces a significant deficit in sensorimotor gating in both adolescent males and females and in adult males as well as compulsive burying behavior in adolescent male rats, while no effects were observed on spontaneous motor behavior. Importantly, a protective effect of communal nesting was observed on both sensorimotor gating deficit and compulsive burying behavior, with significant differences between sexes (Figure 8).

Although early-life isolation did not alter the animals’ spontaneous locomotor activity, we observed a significant effect of sex in two out of three parameters investigated during adolescence and adulthood. With respect to males, female Wistar rats spent more time in the center zone of the open field and travelled a greater distance in the arena (Knight et al., 2021), suggestive of decreased anxiety level and increased motor activity, respectively. Further, when compared to corresponding SH groups, a positive (but not significant) trend can be observed in the time spent in the center of the arena by adolescent CN females, suggesting that a pre-weaning social enrichment condition may decrease the level of anxiety. These findings are in line with previous studies showing that when reared in CN conditions, NIH Norway rats were more likely to enter and spend more time in the center of the open field (Martinez et al., 2015), and that Balb/c mice displayed reduced anxiety-like behavior in the same test (Curley et al., 2009). However, other studies suggested the opposite, with CN reducing in Sprague–Dawley rats the time spent in the open arms in the elevated plus maze and the latency to escape from the light side of the chamber in the light/dark test (Connors et al., 2015), and increasing thigmotaxis in the open field in CD1 Swiss-derived (ICR) mice (Branchi and Alleva, 2006). Such discrepancies highlight the need for further characterization of the effect of pre-weaning social enrichment on emotional behavior, with particular attention given to potential underlying sex- and strain-dependent differences.

Deficits in sensorimotor gating are a transdiagnostic marker of several psychiatric disorders, including schizophrenia (Braff and Geyer, 1990), Tourette syndrome (Castellanos et al., 1996), and bipolar disorder (Perry et al., 2001). PPI deficits are typically modeled in rats by individual housing from weaning until adulthood (Roncada et al., 2009), and these deficits can be reversed by the administration of antipsychotic drugs (Varty and Higgins, 1995). Importantly, gentle handling and environmental enrichment reverse PPI deficits in Sprague–Dawley rats (Varty et al., 2000; Krebs-Thomson et al., 2001), as well as other behavioral and endocrine responses related to early-life stress and psychiatric conditions in Lister Hooded rats (Schrijver et al., 2002), suggesting that social enrichment might also reduce PPI deficits induced by early-life social isolation. The impact of social isolation in the early phase of rearing on sensorimotor gating function has been seldom investigated. Isolation protocols have been typically designed to deprive rodents of social contacts soon after weaning, with male and female Sprague–Dawley rats (Weiss et al., 2001), male Lister Hooded rats (Geyer et al., 1993) and male 129 T2 and C57BL/6 J (Varty et al., 2006) socially isolated immediately after weaning (PND 21–23) and tested on PPI after about 8 weeks (PND 77–80), corresponding to yong-adult age. However, the effect of this environmental manipulation on PPI performance has yielded contrasting findings, depending on several methodological factors, such as timing of rearing, strain, caging condition, protocols, and number of PPI sessions (Geyer et al., 1993; Wilkinson et al., 1994; Domeney and Feldon, 1998). To our knowledge, only one study applied a pre-weaning social isolation paradigm to evaluate its consequences on PPI in male and female Sprague–Dawley rats (Weiss et al., 2001), but in this study the social isolation continued after weaning effectively preventing the assessment of the pre-weaning social isolation per sé on PPI. Interestingly, the developmental timing of the onset of social isolation appears to be a key factor in shaping PPI, as it has been observed that male Sprague–Dawley rats deprived of social contacts especially at adulthood do not exhibit PPI disruption (Varty et al., 1999). Here, we focused on isolation rearing only during the pre-weaning period and found that this protocol elicited a robust PPI deficit which was evident during both adolescence and adulthood in males but only during adolescence in females, suggesting a sex-dependent developmental origin of this environmental model with regard to psychiatric disorders featuring sensory gating impairments (Geyer, 2006). Notably, this study shows for the first time that the PPI deficits induced by early-life social stress were fully prevented by the CN condition in both adolescent and adult males but not in the adolescent females. CN is a naturalistic form of social environmental enrichment that begins from birth until weaning, providing pups with enhanced social and physical, and sensorimotor stimulation. As such, the observed rescue effects of CN in our study might align with the beneficial effects of repeated handling in preventing deficits in prepulse inhibition caused by isolation rearing in Sprague–Dawley and Wistar rats (Krebs-Thomson et al., 2001; Rosa et al., 2005). This suggests that, like repeated handling, CN can also have beneficial effects in mitigating the negative consequences of isolation rearing on sensorimotor integrity during development (Bakshi and Geyer, 1999).

Digging (i.e., the displacement of a substrate using mostly the forepaws), burrowing (i.e., the construction of tunnels for habitation), and burying (i.e., the displacement of either aversive or non-aversive objects underneath any available substrate) are core components of the normal behavioral repertoire of rodents (de Brouwer et al., 2019). The marble burying test is commonly used in preclinical research for the identification of anxiety- or compulsive-like features (Albelda and Joel, 2012). Maternal conditions occurring before and during pregnancy have been linked to the risk of developing obsessive-compulsive disorder (OCD) (Arnold et al., 2018; Mahjani et al., 2020), supporting the evidence that patients with anxiety, OCD and other mental disorders present a history of childhood trauma more often than the healthy population. Further, maternal separation has been reported to increase compulsive burying activity in adult male BALB/c mice, without affecting locomotor activity (Jarrar et al., 2022), and in adolescent but not adult Sprague–Dawley rats, with larger effects in males than females (Abraham et al., 2023). Notably, individual housing soon after weaning did not alter burying activity in young adult Long Evans rats, with isolated males burying a slightly (not significant) higher number of marbles than isolated females (Kinley et al., 2021). This study examined for the first time the effect of the exposure to pre-weaning social stress on burying behavior in rats reared in standard or communal nesting condition, revealing a significant effect of both ESI and housing condition during adolescence but not adulthood. Specifically, while ESI significantly increased burying activity in SH adolescent males, CN fully reverted this effect, without showing an intrinsic effect per. Again, a sex-dependent effect of ESI was detected also in this test, with SH-ESI (but not CN-ESI) adolescent females burying a significantly lower number of marbles than corresponding male group.

Due to the numerous variables involved in this study, i.e., housing condition (SH vs. CN), early-life stress (CTRL vs. ESI), sex (M vs. F) and age (adolescence vs. adulthood), to reduce complexity in the analysis of data we evaluated the effect of the first 3 variables separately in adolescent and adult animals. Yet, a simple comparison of adolescent vs. adult animals in each test shows that (i) the effect of sex in the locomotor activity test observed during adolescence is long-lasting, since it is still evident during adulthood, (ii) the CN condition protects against ESI-induced PPI deficits in males at both ages, (iii) the effect of ESI, CN and sex on burying behavior during adolescence does not persist into adulthood.

Overall, our findings show for the first time that early social experiences and housing condition interact in modulating motor, cognitive and emotional functions in a sex- and age-dependent manner. Further studies are needed to assess the potential benefits of a socially enriched rearing environment in males and females. Questions remain regarding whether being exposed to a socially stimulating environment from birth may effectively protect, or at least attenuate, against other behavioral alterations induced by social isolation early in life.

## Data availability statement

The raw data supporting the conclusions of this article will be made available by the authors, without undue reservation.

## Ethics statement

The animal study was approved by Organismo preposto al benessere degli animali (OPBA), Cagliari. The study was conducted in accordance with the local legislation and institutional requirements.

## Author contributions

JB: Data curation, Investigation, Methodology, Software, Visualization, Writing – original draft, Writing – review & editing. MC: Data curation, Formal analysis, Investigation, Methodology, Software, Visualization, Writing – original draft, Writing – review & editing. AP: Data curation, Investigation, Methodology, Resources, Visualization, Writing – original draft. GT: Data curation, Investigation, Methodology, Writing – original draft, Writing – review & editing. RF: Data curation, Investigation, Methodology, Resources, Writing – original draft, Writing – review & editing. PP: Conceptualization, Data curation, Investigation, Methodology, Writing – original draft, Writing – review & editing. MD: Supervision, Validation, Writing – review & editing, Conceptualization, Data curation. FF: Conceptualization, Funding acquisition, Project administration, Supervision, Validation, Writing – review & editing. PR: Conceptualization, Funding acquisition, Project administration, Supervision, Validation, Writing – review & editing. LR: Data curation, Formal analysis, Software, Visualization, Writing – review & editing. VT: Conceptualization, Funding acquisition, Project administration, Supervision, Validation, Writing – review & editing. RC: Conceptualization, Funding acquisition, Project administration, Supervision, Validation, Writing – review & editing. FS: Formal analysis, Methodology, Software, Validation, Visualization, Writing – original draft, Writing – review & editing. LF: Conceptualization, Funding acquisition, Investigation, Methodology, Project administration, Resources, Supervision, Writing – original draft, Writing – review & editing.
